# A bite so sweet: the glycobiology interface of tick-host-pathogen interactions

**DOI:** 10.1186/s13071-018-3062-7

**Published:** 2018-11-14

**Authors:** Pavlina Vechtova, Jarmila Sterbova, Jan Sterba, Marie Vancova, Ryan O. M. Rego, Martin Selinger, Martin Strnad, Maryna Golovchenko, Nataliia Rudenko, Libor Grubhoffer

**Affiliations:** 1 Institute of Parasitology, Biology Centre, Czech Academy of Sciences, Branišovská 31, CZ-37005 České Budějovice, Czech Republic; 2 Faculty of Science, University of South Bohemia, Branišovská 1760, CZ-37005 České Budějovice, Czech Republic

**Keywords:** Tick, Pathogen, Host, Glycan, Lectin, Glycobiology, *Borrelia*, *Anaplasma*, TBEV, Carbohydrate-binding

## Abstract

Vector-borne diseases constitute 17% of all infectious diseases in the world; among the blood-feeding arthropods, ticks transmit the highest number of pathogens. Understanding the interactions between the tick vector, the mammalian host and the pathogens circulating between them is the basis for the successful development of vaccines against ticks or the tick-transmitted pathogens as well as for the development of specific treatments against tick-borne infections. A lot of effort has been put into transcriptomic and proteomic analyses; however, the protein-carbohydrate interactions and the overall glycobiology of ticks and tick-borne pathogens has not been given the importance or priority deserved. Novel (bio)analytical techniques and their availability have immensely increased the possibilities in glycobiology research and thus novel information in the glycobiology of ticks and tick-borne pathogens is being generated at a faster pace each year. This review brings a comprehensive summary of the knowledge on both the glycosylated proteins and the glycan-binding proteins of the ticks as well as the tick-transmitted pathogens, with emphasis on the interactions allowing the infection of both the ticks and the hosts by various bacteria and tick-borne encephalitis virus.

## **Background**

Vector-borne diseases constitute 17% of all infectious diseases in the world [1]. Pathogenic viruses, bacteria, and protozoa are carried by blood-feeding arthropods on just about all the continents and both livestock and people tend to be affected by these. This becomes a large economic burden on the animal health sector and on the public health system of various countries. Ticks are the first among blood-feeding vectors in terms of the number of pathogens that they can transmit. Unfortunately, there are next to no vaccines against the tick-transmitted bacterial and protozoan diseases and very few against tick-borne viruses [2]. The only successful anti-tick vaccine, based on the glycoprotein Bm86 from the cattle tick *Rhiphicephalus microplus*, has been shown to be efficient against ticks of the genus *Rhiphicephalus* and Bm86 homologue vaccines have had some efficiency against at least two species of the genus *Hyalomma*, but this is not the case for other ticks and the pathogens they transmit [3]. The European Centre for Disease Prevention and Control suggests that there will be a rise in tick-borne diseases based on changes in various factors including the environment and socio-economics [4]. Research efforts to combat tick-borne diseases have usually centred, as with most other infectious diseases, on determining the Achilles’ heel of the pathogen. Most endeavours have focussed on understanding host-pathogen interactions, primarily at the vertebrate level. Protein-carbohydrate interactions between the pathogen and the host cell are of primary importance, in terms of attachment and/or invasion of the cell, whether in an invertebrate or vertebrate host. The observation that there is conservation in the protein-carbohydrate recognition strategies can be used as part of novel approaches for intervention [5]. It has been shown that many regulatory mechanisms are mediated by post-translational modifications (PTM). One example of a PTM that regulates protein degradation and signaling in eukaryotes is ubiquitination. Pathogens are known to exploit ubiquitination to infect mammalian cells and it has been shown that the ubiquitination machinery is present in the tick *Ixodes scapularis*. It was identified that the E3 ubiquitin ligase XIAP restricted bacterial colonization of the vector and *xiap* silencing significantly increased tick colonization by the bacterium *Anaplasma phagocytophilum*, the causative agent of human granulocytic anaplasmosis [6].

Over the last decade, there has been a slow increase in the knowledge of vector-host-pathogen interactions which start from the time a pathogen invades the vector within a blood meal and attaches to the tick midgut lumen. Later it traverses to the tick salivary glands and completes its life-cycle by transmission to a new mammalian host during the subsequent tick feeding [2].

Four possible routes that may facilitate pathogen survival and transmission by most arthropod vectors including ticks have been pointed out. These include: (i) pathogen carbohydrate-binding adhesins that attach to receptors in the tick midgut; (ii) the attachment of carbohydrate-binding proteins of the arthropod to the pathogen as part of its innate immunity; (iii) carbohydrate-binding proteins that are soluble and form a link between the pathogen and midgut surfaces; and (iv) the use of co-receptors to enhance the interactions within the vector [5].

**Fig. 1:**
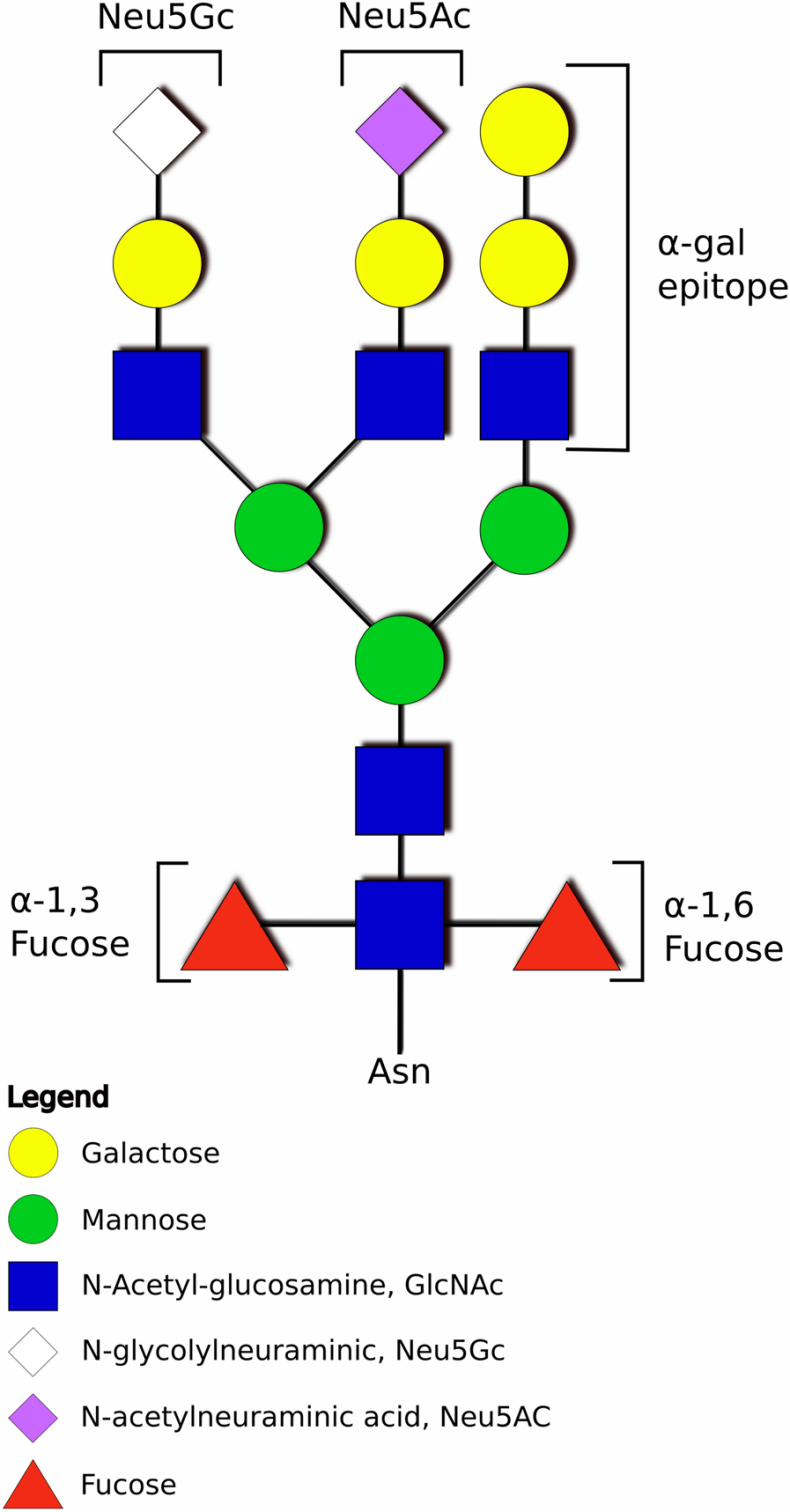
Pathogen-tick and pathogen-host interactions. The scheme represents carbohydrate mediated interactions depicting in particular the most well-known interacting partners of *Borrelia*, tick vector and host (**a**) and *Anaplasma*, tick vector and host (**b**). Both *Borrelia* and *Anaplasma* produce adhesion molecules recognizing either a specific glycoprotein (such as TROSPA, decorin) or a specific glycan (core α1,3-fucose, sialylated glycans) in the tick and the host. Furthermore, *Borrelia* produce proteins interacting with host glycoproteins regulating its immune system. Two examples of the recognized glycans are shown in (**b**): an *O*-glycan bearing both α1,3-bound fucose and also a sialic acid, and an *N*-glycan with its core modified by the α1,3-bound fucose. The used symbol nomenclature is based on the Consortium for Functional Glycomics (http://www.functionalglycomics.org/)

In this review, we would like to highlight the glycobiological aspects of all four of these specific mechanisms that come into play when looking at the vector-pathogen interactions as well as glycobiology-associated processes between the mammalian host and the pathogen (see Fig. 1). Glycobiology of ticks and tick-borne pathogens is developing together with the increased availability and sensitivity of analytical methods; a short overview is listed below, together with some relevant references for readers seeking deeper knowledge.

### **Importance of glycosylation for protein functions**

Post-translational modifications can be found in both prokaryotic and eukaryotic organisms; among them, glycosylation is one of the most abundant and most important. Protein glycosylation affects all the functions of proteins - their structure, activity, interactions with other molecules, half-life in the cell or organism; immune recognition is also dependent on the interaction of immune cells and receptors with glycosylated molecules. A wide variety of possible glycan structures and linkages increases the functionality of proteins [7].

The importance of carbohydrates for the function of proteins can be simply shown on complement proteins. The complement system comprises of more than 30 plasma- or membrane-bound components. Most of them are glycosylated to the various extent and the type of glycosylation generates tissue- or cell-specific population of glycoforms of each complement molecule. The specific glycoform population then secures functions that are required for a particular cell type or tissue. The repertoire of functions secured by glycans ranges from the control of protein folding, proper assembly within the endoplasmic reticulum, mechanistic shielding of the protein backbone against protease degradation, preventing inappropriate protein-protein interactions or formation of proper spatial protein conformation or participation in recognition epitope formation [8]. Specific examples show interesting ways in which glycans influence or modulate the complement cascade.

C1q is a recognition molecule of classical complement pathway and mediates initiation of the pathway by binding to the antibody-antigen complexes. The proper function of the C1q is conditioned by the appropriate triple helix formation within C1q monomer and the formation of C1q hexamer whose spatial conformation may be secured by the presence of N-linked glycan of each monomer [9].

Glycosylation was also proven important in complement regulatory factors where factor H (fH) glycosylation mediates its proper folding within the endoplasmic reticulum (ER). The absence of glycosylation or its malfunctioning leads to in fH misfolding and retention in ER causing clinical symptoms in children in form of hypocomplementemic renal disease [10].

C1-inhibitor is a plasma glycoprotein and, along with other members of the serpin proteases, its inhibitory activities are enhanced by binding of negatively charged polysaccharides. Most of the polysaccharides binding to the C1-inhibitor induce allosteric changes of the inhibitor molecule causing potentiation of the attachment to the C1 proteases or, as in case of dermatan sulphate, the potentiation is caused by the formation of a negatively charged polysaccharide-mediated linkage between positively charged portions of the C1-inhibitor and C1 protease molecules [11]. Structural characterization of the C1-inhibitor reveals extensive O-glycosylation with a high number of sialylated glycans. The trials for functional characterization of C1-inhibitor glycosylation showed an increased resistance of highly O-glycosylated region against proteolytic degradation [12] and highlighted the importance of sialylation for prolonged serum half-life [13].

### **Advances in bioanalytical methods for glycobiology**

The most frequently used methods in glycobiology are mass spectrometry in combination with chromatography or capillary electrophoresis, glycan/lectin microarrays, or lectin staining. All of these developed greatly recently; for example, the increasing number of mass spectrometers available throughout the world and the development of more sensitive instruments and specifically the introduction of the Orbitrap mass spectrometers, greatly advanced the possibilities for glycan and glycoprotein analysis [14, 15]. The number of commercially available microarrays is also increasing and nowadays allows more or less specific detection of almost any kind of glycan. The availability of lectins together with the possibility to synthesize specific glycan molecules allows also the preparation of in-house glycan- or lectin-arrays; another possibility is the service provided by the Consortium for Functional Glycomics (http://www.functionalglycomics.org/).

Here, we review the current knowledge on how pathogens have evolved “sweet” strategies to overcome the immune responses within the vector and the mammalian host and the use of carbohydrate-binding properties to perpetuate their transmission and dissemination into a vertebrate host (Tables 1 and 2). We also provide a near comprehensive catalogue of all carbohydrate molecules that play a part in the disease cycle that have been characterized to date, be it within the tick or the mammalian host. We would like this to be the start of a renewed interest in the glycobiology of ticks and tick-borne diseases.

**Table 1:** Summary of carbohydrate-binding proteins of *Borrelia* spp. recognizing tick or host receptors. The carbohydrate-binding proteins from *Borrelia* spp. are listed together with the recognized molecule from the vector or the host. Glycoproteins or glycans are listed as the recognized molecules depending on the available information. Majority of proteins from Lyme borreliosis spirochetes are listed; in the case of relapsing fever *Borrelia*, the bacterial species is defined

*Borrelia* spp. protein	Tick binding partner	Reference
*Borrelia vs* tick		
OspA	TROSPA	[236]
OspC	SALP15	[27]⁠
TSLPI/P8	Mannose binding lectin (MBL)	[42]
Vsp33 (*B. hermsii*)	Unknown receptor in tick SG	[62]
*Borrelia vs* host		
Bgp (p26)	GAG	[294]
DbpA (p20)	Decorin/dermatansulfate	[48, 295]
DbpB (p19)	Decorin/dermatansulfate/chondroitinsulfate	[48, 295]
Bbk32	Fibronectin /heparansulfate/dermatansulfate	[63]
P66	Integrins	[87]
OspA	Plasminogen	[296]
OspC	Plasminogen	[297, 298]
Enolase	Plasminogen	[299]
Erps (OspE/F related proteins)	Factor H or FHL protein	[105]
CRASPs	Factor H	[105]
PAMPs	Mannose receptor on dendritic cells	[300]
Unknown	Neolacto-(Gal4GlcNAc3Gal4Glc1)-carrying glycoconjugates in human erythrocytes	[301]
VspB (*B.turicatae*)	GAG	[61]

**Table 2:** Summary of carbohydrate-binding proteins of *Anaplasma* recognizing tick or host receptors. The carbohydrate-binding proteins from *Anaplasma* are listed together with the recognized molecule from the vector or the host. Glycoproteins or glycans are listed as the recognized molecules depending on the available information

*Anaplasma* protein	Binding partner	Reference
MSP1a (MSP1 complex)	Vector binding partner: Unknown receptor in IDE8 tick cells	[189, 190]
Unknown molecule	Vector binding partner: Core α 1,3-fucose glycoprotein	[203]
Unknown adhesin-like molecule	Host binding partner: 1,3-Fuc and Sia in sialyl Lewis X, PSGL-1 in human neutrophils	[199, 200]
Unknown adhesin-like molecule	Host binding partner: 1,3-Fuc and Sia in sialyl Lewis X, PSGL-1 in murine neutrophils	[199]
Unknown adhesin-like molecule	Host binding partner: 1,3-Fuc and Sia in sialyl Lewis X, PSGL-1 in human myeloid HL-60 cells	[197, 201]
Unknown molecule of *A. phagocytophilum* NCH-1 strain	Host binding partner: 1,3-fucose in murine bone marrow-derived mast cells (BMMCs), murine peritoneal mast cells	[198]
Unknown molecule of *A. phagocytophilum* NCH-1 strain	Host binding partner: α 1,3-Fuc in human skin-derived mast cells	[198]
AmOmpA	Host binding partner: α2,3-sialylated and α1,3-fucosylated glycan of the sialyl Lewis x in myeloid cells	[182]
AmOmpA	Host binding partner: α2,3-sialylated and α1,3-fucosylated glycan of the 6- sulfo-sialyl Lewis x in endothelial cells	[180, 182]
AmOmpA	Host binding partner: α2,3-sialylated, α2,6-sialylated, α1,3-fucosylated glycan receptors in human and murine myeloid HL-60 cells, 6- sulpho-sialyl Lewis x in endothelial cells	[180–182]
Unknown	Host binding partner: α1,3-fucose	[203]

## **Glycosylation in the*Borrelia*infection cycle**

Compared to eukaryotes, glycosylation in bacteria produces a much more diverse repertoire of glycoconjugate structures which are often species- or strain-specific. Most of the bacterial glycoconjugates are an integral part of the bacterial cell wall and provide the bacterial cell structural integrity. Additionally, the bacterial glycosylated cell surface structures mediate adhesion and interaction with its environment or host. Although the structural features of bacterial surface glycans have been well described, the function of many of them, including those in pathogenic bacteria, remain unexplored. In principle, pathogenic bacteria use glycosylation for two reasons; they synthesize host-like glycan structures to hide from the host immune system and, conversely, they produce glycosylated proteins that are able to bind more effectively the host immune molecules and thus influence their activity [16].

Since all *Borrelia* species are host-propagated bacteria that shuttle between a vertebrate host and an arthropod vector, these spirochetes have developed strategies to adjust to these diverse environments [17]. This is achieved by regulating the level of gene expression in response to changes in temperature, pH, salts, nutrient content, and other host- and vector-dependent factors. A significant number of *Borrelia* proteins mediate the interactions with host/vector molecules and thus enable *Borrelia* to complete its infectious cycle. Recent findings highlight the importance of carbohydrate moieties in these interactions and in the overall pathogenesis of this infectious spirochete.

### ***Borrelia*/tick glycosylated interactions**

When entering the vertebrate host during tick-feeding, *Borrelia* must overcome several barriers to successfully invade and disseminate in the host body. The invasion of the host is difficult as it requires the interaction of the existing *Borrelia* surface structures with host tissues without being noticed by the host immune system. *Borrelia* have developed many elaborate strategies to recognize diverse host molecules and cell types to promote dissemination and chronic infection [18], and to overcome host immune system surveillance [19]. The concerted action of these structurally and functionally diverse *Borrelia* surface molecules helps the spirochete to successfully adapt and multiply in the host body.

The interacting molecules of *Borrelia* and the tick are often modified by glycosylation producing a diverse pool of structures. Moreover, glycosylation is a dynamic modification and can be readily altered upon environmental cues [20].

The presence of glycoconjugates on the surface of cultured *B. burgdorferi* has been demonstrated by the ability of *Borrelia* to bind a number of lectins [21]. In search of *B. burgdorferi* glycosylation patterns, increased attention has been paid to outer surface proteins that are produced at different stages of the *Borrelia* transmission cycle and represent points of interaction between the spirochetes and their hosts/vectors.

### ***Borrelia*outer surface proteins**

*Borrelia* outer surface proteins A and B (*osp*A and *osp*B) are encoded on a bicistronic operon and extensively expressed on the surface of spirochetes in unfed ticks. OspA is one of the major and most comprehensively studied *Borrelia* proteins. While OspA mediates *Borrelia* attachment to the tick midgut when spirochetes are acquired by ticks during blood-feeding, OspB plays a key role in successful colonization of the tick midgut. OspA downregulation is important for *Borrelia* detachment, multiplication, and migration from the tick midgut to salivary glands [22–25]. When ticks are fed to engorgement, *Borrelia* clears OspA and OspB from the surface expressing instead another outer surface protein C (OspC) [22, 26].

OspC, encoded by *bbb19* mapped to the cp26 plasmid, is one of the most divergent genes in *Borrelia* genome, and is crucial for the early stages of mammalian host infection by the spirochete, but not required for acquisition of spirochetes by tick, tick colonization or migration from salivary glands to the gut [27–32].

Erps (OspE/F-related proteins) are a family of surface integrins with high affinity to factor H and encoded by *erp*-loci localised on each of the cp32 plasmids. Lyme disease spirochetes control Erp synthesis throughout the bacterial infectious tabacycle, producing the proteins during the infection of the host but downregulating their synthesis during tick infection stage. The best-characterized members are OspE and OspF proteins [33], their paralogues OspE/F-related proteins [34] and a group of OspE/F-like leader peptides (Elps) [35].

#### ***OspA, OspB and TROSPA***

Earlier work had indicated that OspA and OspB are the major *Borrelia* glycosylated proteins [36], yet a later study showed that the suggested *N*-linked glycosylation does not occur [37]. Colonization of ticks by spirochetes requires the involvement of tick receptor(s). Although a tick receptor for OspB has not yet been identified, the tick receptor for OspA (TROSPA) is located in the tick gut and is heavily glycosylated. The blockade of TROSPA by TROSPA antisera or by downregulation of TROSPA *via* RNAi reduced *B. burgdorferi* adherence to the tick gut, hampering the spirochete transmission to the mammalian host. The number of potential posttranslational modification sites in TROSPA is unusually high (> 30), with a predominance of *O*-glycosylation sites [25].

#### ***OspC and Salp15***

When transmitted from the tick vector to the host, *Borrelia* are delivered within the tick saliva. Tick saliva contains a plethora of bioactive molecules, which have been shown to be important for immunosuppression of the host responses [28]. One of the secreted salivary proteins is *Salp15* [29]. This protein specifically interacts with *B. burgdorferi* OspC which results in the protection of *Borrelia* from antibody-mediated killing and plays a critical role in establishing *B. burgdorferi* infection [27]. Whereas no data exists about the potential glycosylation of OspC, the glycosylation of Salp15 was demonstrated experimentally [30]. Salp15 from *I. ricinus* did not deliver the same protection to *B. garinii* and *B. afzelii* against antibody-mediated killing [31], presumably suggesting that the Salp15 binding for some species is an advantage for surviving in nature [31]. An explanation may lie in a different structural or spatial organization of the OspC or Salp15 molecule causing better access to the binding sites of each of the molecules in *B. burgdorferi.* Another hypothesis claims that *B. burgdorferi* OspC holds differently charged areas which interact in a way that favour formation of OspC multimers or even a lattice [38]. This structure, along with bound Salp15, might form a protective coating on the bacteria preventing an access of anti-OspC antibodies or *B. burgdorferi* antiserum [27]. In addition to the direct interplay between Salp15 and *B. burgdorferi*, Salp15 indirectly facilitates the host invasion by inhibiting dendritic cell activation by binding to the receptor/lectin DC-SIGN, localized on the surface of macrophages and dendritic cells [32].

#### ***Ixofin3D and Ixodes scapularis dystroglycan-like protein***

Ixofin3D and *I. scapularis* dystroglycan-like protein (ISDLP) are glycoproteins expressed on the surface of midgut cells which were identified as candidate tick midgut binding partners of *B. burgdorferi* using a yeast surface display assay [39]. The expression of both Ixofin3D and ISDLP was elevated in *Borrelia*-infected tick midgut during feeding. Ixofin3D and ISDLP interact with spirochete cells as was confirmed *in vitro* by immunofluorescence assay and RNA interference. The RNAi-mediated reduction in expression of Ixofin3D and ISDLP resulted in decreased spirochete burdens in the tick salivary glands and in the murine host as well [39, 40]. The full-length Ixofin3D contains four putative fibronectin type III domains. Ixofin3D is glycosylated as shown experimentally by periodic-acid Schiff’s staining of a recombinant protein produced in *Drosophila* cells. Even though the importance of Ixofin3D for *Borrelia* infection was shown, the borrelial binding partner for Ixofin3D has not yet been identified [39].

The binding partner for ISDLP is also yet to be discovered. Like Ixofin3D, ISDLP silencing did not reduce the spirochete numbers in the gut but the transmigration process from gut to salivary glands was impaired. The mechanism remains unknown although the collected evidence implies that ISDLP may facilitate gut tissue remodelling or reduced barrier for spirochete transfer to salivary glands [40].

#### ***TSLPI***

Tick salivary lectin pathway inhibitor (TSLPI) is a secreted salivary protein that protects *Borrelia* from complement-mediated killing. TSLPI facilitates spirochete transmission and acquisition through interference with the host mannose-binding lectin (MBL) and inhibition of the host lectin complement pathway [41]. *N-*linked glycosylation of recombinant *Drosophila*-expressed TSLPI appeared to be vital for its function as a lectin pathway inhibitor, suggesting that TSLPI *N-*glycans are involved in its binding to MBL carbohydrate recognition domains [42, 43].

### ***Borrelia*adhesins and extracellular matrix**

Adhesion is the first and basic event in establishing an infection. The *Borrelia* cell surface is, at the time of host invasion, covered by adhesion proteins that can recognize and bind to various host cell types and/or extracellular matrix (ECM) components and thus promote *Borrelia* dissemination and settlement in various corners of the host body. Although *Borrelia* adhesins are not glycosylated, the presence of glycosylation has been confirmed in their tick receptors, suggesting a significant role of glycosylation in adhesin-receptor interaction.

*Borrelia burgdorferi* encodes a variety of adhesins and their characterization and role in the *Borrelia* infection cycle using different approaches was thoroughly described in a review by Coburn et al. [44]. With regard to their overlapping roles in *Borrelia* adhesion to the host tissue, it is important to note that only the concerted action of various adhesins guarantees an effective adhesion and transmigration of spirochetes to different hosts and their tissues [44].

## **A short overview of host ECM proteins**

### **Glycosaminoglycans**

Several ECM-associated molecules are specifically targeted by *Borrelia* adhesins. Exceptionally important seem to be the glycosaminoglycans (GAGs), large linear polysaccharides constructed of repeating disaccharide units (e.g. hyaluronan, chondroitin, dermatan, heparan, keratan) that decorate the ECM proteins. GAG chains are abundantly modified by sulphurylation, which imparts them a strong negative charge [45]. Numerous studies have shown that binding of GAGs by *B. burgdorferi* enables colonization of the host [46].

### **Fibronectin**

A relevant ECM-associated molecule for *B. burgdorferi* attachment is fibronectin (Fn), a high-molecular weight, dimeric glycoprotein found in body fluids and in the ECM. *Borrelia burgdorferi* fails to bind to the ECM *in vitro* upon exposure to an anti-Fn antibody, implicating the Fn involvement in *Borrelia* attachment [47].

### **Decorins**

Decorins are ubiquitous ECM proteoglycans, which are associated with collagen fibrils in the mammalian connective tissues [48]. Decorins are complex glycoproteins; apart from serine linked GAG chain, they are also modified by up to 3 N-glycans [49, 50]. Numerous studies associate decorins with *Borrelia* adhesin attachment and interestingly, the binding is promoted by intact decorin proteoglycan molecule rather than its protein core or GAG chain itself [48].

### **Laminin**

Laminin is a large extracellular matrix multidomain glycoprotein. It is a critical molecule in the basement membrane assembly and, by extension, in tissue formation in the developing organism [51]. Besides its role in basement membrane architecture, it also mediates cellular interactions and provides a dense network for various cellular signalling and attachment events. The existence of different laminin isoforms gives space to developmental regulations mediated by differential responses to cells and newly forming tissues. Laminin, as well as other ECM forming molecules, possesses numerous glycosylation sites and its molecule is modified up to 32 % by N-linked glycans [52].

The carbohydrate portion of Laminin was proven to be a mediator of attachment in several bacterial species [53]. Laminin is also a potent target of several borrelial adhesins [54–56] although the direct involvement of the laminin carbohydrate moiety has not been reported yet.

### **Integrins**

Integrins are glycosylated cell surface receptors mediating cell adhesions to the extracellular matrix and some important cell-cell interactions [57]. The presence of *N-*linked glycans in integrin molecule proves integral for the stability of the domain conformation and consequently affects integrin adhesive properties [58]. Integrins possess a typical heterodimeric structure combining different α and β polypeptide chains and their combination determines the specificity of integrins [59]. Integrins are expressed on all mammalian cells except erythrocytes. The expression of different integrin subtypes produces unique cell surface signature of each cell type [60].

### ***Borrelia*adhesins**

#### ***Vsps***

Relapsing fever *Borrelia*, unlike Lyme disease-causing *Borrelia*, are vectored by soft ticks of the genus *Ornithodoros*. They are present in the blood of the mammalian host in high numbers which lead to high fevers followed by bouts of relapses. They recognize glycosaminoglycans (GAG), which mediates the attachment of *Borrelia* to mammalian cells. GAG recognition is partly dependent on the presence of some of the variable small proteins (Vsps). *Borrelia turicatae*, a relapsing fever borrelia that is vectored by *O. turicata*, recognizes GAGs *via* VspB which allows binding of *B. turicatae* to cultured mammalian cells as well as increased spread and replication in the mammalian host. *Borrelia hermsii* also attaches to cultured mammalian cells *via* GAGs; however, Vsps are not essential for this binding [61].

After the feeding of *O. hermsi* with *B. hermsii-*infected blood, the bacteria switched from expression of many bloodstream outer surface variable major proteins (Vmps) to a unique protein, variable tick protein (Vtp, Vsp33) [62].

#### ***BBK32, RevA and C1-inhibitor***

*Borrelia burgdorferi* expresses at least two fibrinogen-binding proteins, BBK32 [63] and RevA [64]. BBK32 is a protein, whose attenuation does not block the spirochete transmission from the tick to the host [65], but lowers the bacterial loads in different tissues at different time points of infection [66]. *Borrelia burgdorferi* also attaches to endothelium in the vascular system through fibrinogen (Fn) and this interaction becomes stronger with increasing blood flow, allowing the spirochete to overcome fluid shear stress [67]. These stabilizing interactions are sustained by catch bond properties of BBK32 [68]. Following the binding to Fn, BBK32 binds to various kinds of GAGs, including heparin sulphates and dermatan sulphates of the host ECM [69–71]. It also seems to be involved in the modulation of the innate immunity. In particular, BBK32 binds the C1 complex of the classical innate immunity pathway, preventing its activation and thus obstructing classical pathway-mediated *Borrelia* lysis [72].

As *B. burgdorferi* BBK32 mutants are still able to bind fibronectin, an additional Fn-binding protein, RevA, was identified [58]. RevA expression on the *Borrelia* cell surface was upregulated in mammalian host compared to the tick vector. Furthermore, *Borrelia-*infected patients produced anti-RevA antibodies throughout various stages of Lyme disease suggesting its involvement in Lyme disease establishment and persistence in the host. RevA appears to have multiple binding sites which *Borrelia* uses to bind host cells *via* Fn [46, 73].

#### ***DbpA/DbpB***

Decorin-binding proteins A and B (DbpA and DbpB) are adhesins found on the surface of *B. burgdorferi* [20, 45]. These proteins are critical for the virulence of *B. burgdorferi* [74, 75]. New data suggests that the decorin-binding proteins actually do not bind directly to the decorin protein core but interact with decorin *via* GAGs that are attached to the protein [76–78]. The binding studies of DbpA and DbpB from different *Borrelia* genospecies showed that there are clear differences in the decorin binding activity and that these differences may ultimately lead to the differences in tissue tropism and clinical manifestations associated with particular *Borrelia* genospecies [76, 79]. *In vivo* functional studies demonstrated the importance of DbpA/B adhesins for *Borrelia* invasion of the mammalian host especially in the early stages of infection [80].

#### ***Bgp***

*Borrelia burgdorferi* glycosaminoglycan binding protein (Bgp) is a surface-exposed protein on intact spirochetes [70]. Recombinant Bgp bound the same GAG as the whole spirochete, agglutinated erythrocytes and inhibited binding of *B. burgdorferi* to the mammalian cells. A transposon mutant of the *Bgp* gene had less ability to adhere to host endothelial and epithelial cells *in vitro* and to colonize host target tissues leading to the reduced inflammatory manifestation of Lyme disease in the mouse model. The adherence was not fully disrupted due to the existence of other GAG-binding adhesins which facilitate host colonization and also highlights the importance of *Borrelia* GAG-binding ability for the completion of the infection cycle [81]. Although the Bgp attachment to GAG is not essential for disease establishment, the protein appears to be involved in the formation of an initial infectious niche in the host. Different spirochetes strains possess different GAG-binding preferences and their binding ability to multiple cells depends on the GAGs that they express [76].

#### ***BmpA***

BmpA (*Borrelia* membrane protein A) and its three paralogues B, C, and D are all laminin-binding borrelial outer surface proteins [82]. Like other *Borrelia* surface proteins, BmpA is also antigenic. All bmp genes are located on the *Borrelia* chromosome, arranged in clusters that are differentially regulated [83]. The involvement in the development of arthritis in the mouse model was described for two Bmp proteins, BmpA and BmpB [84].

### ***Borrelia*adhesins and integrin-mediated interactions**

*Borrelia* binds to host endothelial cells *via* the interaction of integrins α_IIb_β_3_, α_V_β_3_, and α_V_β_1_ with *Borrelia* surface adhesins [59, 85]. It was also described that the causative agent of relapsing fever, *B. hermsii*, binds to human platelets promoted by the platelet glycoprotein integrin α_IIb_β_3_ and is diminished by α_IIb_β_3_ antagonists or by a genetic defect in this integrin [86].

#### ***P66***

P66 is one of the candidate ligands for β_3_-chain integrins (e.g. α_IIb_β_3_, α_V_β_3_) [87]. P66 also functions as a porin [88, 89], and structural predictions, as well as some experimental data, present the molecule as porin assuming the structure of β-barrel [90].

P66 mutants showed a dramatically reduced ability to attach to integrin α_V_β_3_ [91]. Endothelial cells responded to wild-type *Borrelia* infection by upregulation of endothelial growth factor compared to a control infection with a P66 deletion mutant. The ability of P66 mutants to transmigrate through the cell monolayer was impaired, which suggests the role of P66 in *Borrelia* transendothelial migration, although its porin function does not play a role in the migration process [92].

Mammalian integrins typically contain an RGD (Arg-Gly-Asp tripeptide) consensus sequence in their binding domain, where aspartic acid is a key binding amino acid. P66 lacks this sequence; however, residues 205 and 207 of its 203–209 binding region are both aspartic acid [93]. P66 deletion mutants applied subcutaneously are readily cleared out of the site of infection, which refers to the possible involvement of the innate immune system and confirms the importance of this protein for host colonization together with other studies [94]. However, tick colonization is shown to be P66-independent [94].

#### ***BB0172***

BB0172 is an outer membrane protein containing von Willebrand factor A domain which mediates intercellular and protein-protein interactions in ECM. It is, for example, involved in the attachment of platelets to the ECM in the site of damaged endothelial epithelium *via* platelet surface glycoprotein [95]. BB0172 showed a weak interaction with ECM-associated fibronectin. Importantly, a strong affinity was observed in the attachment of BB0172 to α_III_β_1_ integrin. Moreover, the affinity was much stronger than the one observed in the interaction of borrelial P66 adhesins with β_3_ chain integrins [95].

### ***Borrelia*adhesins interacting with mammalian complement**

Mammalian innate immunity is alerted by a variety of surface-exposed molecules of invading pathogens. The first encounter of host antibodies with potentially harmful intruder activates the complement system which assists in tagging of the pathogen for destruction and also acts on pathogen clearance itself by the formation of membrane attack complex. Different pathways of the complement system progress in a cascade-like manner and its brisk response to pathogen invasion must be under the control of regulating mechanisms preventing complement from attacking host cells.

Invading a host organism, the pathogens have evolved different strategies to circumvent the immune response. Many of these strategies are in fact directed against components of the complement system. The most widespread strategy employs molecules recruiting or mimicking the complement regulators, including the direct interaction of pathogens with complement proteins leading to the modulation or inhibition of their function or indirectly to the activation of complement proteins enzymatic degradation [96].

The complement regulators are represented by several serum proteins that are able to dampen the activity of complement and prevent host self-destruction. Two of them, complement factor H (FH) and its splice homologue Factor H-like (FHL) inhibit the alternative complement pathway response using host-|specific surface patterns like sialic acid or GAGs and thus promoting self-recognition processes [97, 98].

FH is a plasma glycoprotein containing 9 glycosylation sites [99] bearing complex, predominantly diantennary disialylated, fucosylated, and nonfucosylated glycans at eight of the nine glycosylation sites [100]. Similarly, FHL is also a plasma glycoprotein [101]. Both proteins possess a RGD motif which is assigned cell adhesive properties and thus can modulate cell adhesion. Additionally, FHL promotes anchorage-dependent cell attachment and spreading [102].

#### ***CRASPs and ERPs***

The two complement regulators, FH and FHL are bound by *Borrelia* surface proteins hence preventing the activation at the central step of the complement cascade. Serum resistant *Borrelia* express adhesins on their surface, which are capable of interfering with different components of the host complement system leading to the modulation of host immune response and hampering the complement-mediated spirochete lysis [103, 104].

The two well-characterized types of complement interfering adhesins, complement regulator-acquiring surface proteins (CRASP) 1 and 2 [105], control the complement activity by binding complement regulating molecules such as FH and FHL-1 [104, 106]. Up to now, five different CRASPs (CRASP-1 to CRASP-5) have been described and each of them presents a different binding ability to FH, FHL-1, or plasminogen [98, 104, 106].

CRASP-1 (CspA, BBA68) has been studied the most extensively. It shows a strong affinity to the complement regulators which inactivate the complement response very efficiently [106, 107].

The expression of CRASP-1 is repressed in the tick vector and increases in the mammalian host, which suggests its role in spirochete transmission and evasion of the host immune response [108, 109]. CRASP-1 also confers serum resistance to *B. burgdorferi*. The role of CRASP-1 in complement inactivation is evident in the CRASP-1 knockout-mutants which inefficiently bound human FHL and attracted complement constituents more readily [110, 111].

Apart from *B. bavariensis*, all studied *Borrelia* species possess CRASP-1 orthologues conferring complement inactivation [112, 113]. The orthologues belong to the same protein family although the encoding genes do not share the same locus with the *B. burgdorferi* CspA [98].

CRASP-2 (CspZ) is another *Borrelia* adhesin binding both FH and FHL-1 independently of CRASP-1 and reinforcing *Borrelia* complement resistance [114, 115]. The CRASP-2 expression fluctuates in a somewhat similar manner to CRASP-1 during the *Borrelia* infectious cycle. Like CRASP-1, CRASP-2 is also upregulated during an established mammalian infection and is able to activate antibody-mediated immune response [116], which makes this adhesin important for the diagnosis of Lyme disease infection. The triggered immune response does not, however, provide the host with protective immunity and has no effect on spirochete dissemination [117].

Three members of the polymorphic Erp (OspE/F-related protein) protein family, ErpA (BBP38, CRASP-5), ErpC (CRASP-4) and ErpP (BBN38, CRASP-3), are plasminogen binding proteins that can simultaneously bind to FH and FH-related proteins [103, 107, 118–122].

The Erp proteins are most probably involved in different reservoir hosts infection due to differential binding abilities of particular Erp paralogues [123, 124]. Despite their complement regulator binding properties, none of the Erp proteins are necessary for the protection of *Borrelia* spirochetes against complement-mediated killing; CRASP-1/CRASP-2 deletion mutants expressing all Erp proteins were susceptible to serum mediated lysis [119, 120, 125].

Erps are not upregulated during *Borrelia* transmission but their expression gradually increases during Lyme disease progression, suggesting their role during mammalian infection [125]. Interestingly, *Borrelia* can regulate the expression of both Erps and CRASPs very dynamically as different isolates of *B. burgdorferi* (*s.l.*) reacted differently to complement-mediated killing [56, 126]. Moreover, some of the Erp members present multiple functions during *Borrelia* infection. For example, ErpX ability to bind complement regulators is complemented by its laminin binding properties [56]. The overlapping activities of *Borrelia* surface molecules enhance the overall infectious potential of the spirochete.

### ***Borrelia*-specific host pattern-recognition receptors and lectins**

#### ***Toll-like receptors***

Recognition of pathogens is mediated by a set of pattern-recognition receptors (PRRs). The group of glycosylated proteins that comprise the Toll or Toll-like receptors family (TLRs) are transmembrane receptors that function as PRRs in mammals [127]. So far, eleven members that potentially participate in the recognition of invading pathogens have been identified in mammalian genomes [128] and glycosylation was shown to have a critical role in TLR presentation on the cell surface [129, 130].

There are several TLR members, whose role in spirochete recognition has been identified. The well-characterized TLR2 is presented on antigen-presenting cells, epithelial and endothelial cells [131]. It was able to recognize a variety of ligands and was important for macrophage activation and further triggering of the immune response in *Borrelia-*infected mammalian hosts when stimulated by OspA [132]. The signal transduction through TLR1/2 in response to *B. burgdorferi* invasion can elicit opposite immunoregulatory effects in the blood and CNS immune cells, affecting the different susceptibility of these compartments to infection [127].

TLR4 is expressed in macrophages and dendritic cells [130] and is upregulated upon *Borrelia* infection or stimulation by OspC [133, 134] and its main ligands are lipopolysaccharides (LPS) from gram-negative bacteria [135]. The role of TLR4 in *Borrelia* recognition remains unclear as *B. burgdorferi* does not express LPS on its surface.

TLR9 is responsible for recognition and further endosomal/lysosomal internalization of CpG motifs in bacterial DNA [136]. This process has been observed in sonicated *Borrelia*, which promoted the activation of murine cells *via* TLR9 [137].

#### ***Nucleotide-oligomerization domain-like receptors***

Nucleotide-oligomerization domain-like receptors (NOD-like receptors or NLR) are a group of intracellular PRRs, capable of binding bacterial muropeptides, the molecules derived from bacterial peptidoglycans [138]. Together with TLRs, NOD-like receptors are crucial for recognition of *Borrelia* species. Contrary to other PRR families, NLRs bind bacterial ligands intracellularly, i.e. they are able to recognize the pathogen-associated molecular patterns (PAMPs) that enter the cell *via* phagocytosis or through the membrane pores induced during cellular stress [139].

There are several NLR protein members that can bind carbohydrate-associated PAMPs, although only a few of them were directly observed to be involved in Lyme disease. NOD1 and NOD2 receptors are the most extensively investigated major PRRs [138, 140].

*Borrelia-*infected primary murine astrocytes upregulated NOD-proteins upon exposure to some TLR-ligands [138], while murine primary microglia infected by *Borrelia* only upregulated NOD2 and not the NOD1 [141]. NOD2 activation by *Borrelia* stimulated inflammatory cytokines release. Their activities are assigned to a host proinflammatory response, although their particular role in Lyme disease establishment remains unknown [142]. NOD2 stimulation by *Borrelia* induces inflammation during the early stages of Lyme disease but induces tolerance and suppresses *B. burgdorferi-*mediated Lyme arthritis and carditis in mice during later phases of infection [143]. *Borrelia* recognition in the host is conferred by the combined action of TLR and NOD2. The activation of both receptors at a time by *Borrelia* species is essential for an effective cytokine release. It has been concluded that TLR2 and NOD2 co-recognition of *Borrelia* surface receptors leads to both induction of a proper immune response and to inflammatory-induced pathology [144].

#### ***C-type lectin receptors***

A family of calcium-dependent receptors that bind carbohydrate ligands include both soluble and cell-associated (transmembrane) lectins in vertebrates. C-type lectin receptors (CLRs) expressed by dendritic cells are crucial for tailoring immune response to pathogens. The transmembrane type is predominantly expressed by antigen-presenting cells functioning as PRRs recognizing PAMPs in bacteria [128]. Currently, 17 CLR subfamilies are described in vertebrates.

Mannose receptor represents a subgroup of CLRs binding mannose-containing bacterial transmembrane PAMPs. CLRs are involved in the recognition and phagocytosis of several microorganisms including *B. burgdorferi*. In particular, CLRs were upregulated in dendritic cells after *B. burgdorferi* activation and facilitated phagocytosis of *B. burgdorferi* by monocytes and macrophages [128]. However, the recognized borrelial protein is yet to be identified.

### **Surface glycolipids of*Borrelia burgdorferi***

*Borrelia* have an unusual composition of glycolipids in their outer membrane; they synthesize mono-α-galactosyl-diacylglycerol (MGalD) and cholesterol derived glycolipids cholesteryl-β-D-galacto-pyranoside, cholesteryl 6-O-acyl-β-D-galactopyranoside (ACG), or cholesteryl 6-O-palmitoyl-β-D-galactopyranoside (ACGal/BbGL-1) [145–147].

The *Borrelia* glycolipids induce inflammatory reactions; in particular, two glycolipids ACGal/BbGL-I and MGalD/BbGL-II, are probably immunogenic [145, 148]. The immunogenic epitope is recognized in the lipid part of the glycolipids [149]. An important constituent of the immunogenic epitope is the α-linked terminally bound galactose which is recognized by the T-cell receptor of invariant natural killer T cells (NKT) [150]. This then promotes their activation as well as the proliferation of Lyme disease-directed antibodies [151–153] which recognize glycolipids in the cell membrane of *Borrelia* but also *Ehrlichia* [154]. Importantly, the induced antibodies against the glycolipid fraction cross-react with gangliosides, which explains the phenomenon of neuroborreliosis [155].

The glycolipid recognition by invariant NKT cells seems to be an alternative system for innate immune system activation by bacteria lacking LPS, an otherwise typical antigenic determinant of most gram-negative bacteria [156].

*Borrelia* bind to GalCer (galactosylceramide) on Schwan cells [157], LacCer (lactosylceramide), ceramide trihexoside and gangliosides GD1a and GT1b. Moreover, *Borrelia* displays a specific affinity to disialoganglioside GD1a and trisialoganglioside GT1b carrying sialic acid. The ability to bind such a wide range of glycosphingolipids might provide an explanation for its ability to adhere to a wide spectrum of different cell types [158]. *Borrelia* did not bind gangliosides GM1, GD1b, GM2, GM3 and asialo-GM1 implying the requirement for terminally bound sialic acid in ganglioside recognized epitope and demonstrates the specific character of *Borrelia* and acidic gangliosides interaction. Interestingly, adhesion to GD1a and GT1b, as well as GalCer or LacCer was not compromised by free sialic acid, galactose or lactose, respectively [158, 159]. Conversely, GalCer-binding sites were saturable using free GalCer in CHO-K1 cells preventing spirochetes from attachment [148].

## **Vector-host glycosylated interactions**

Similarly to *Borrelia*, the tick’s successful evasion of the host response depends on its ability to conceal its activities from the host immune system. The pursuit of successful feeding drove ticks to equip their saliva with multiple pharmacologically active molecules which feature immunomodulatory activities. The myriad of diverse functions include cytolysis, vasodilatation, anticoagulation, anti-inflammation and immunosuppression. The comprehensive list of tick pharmacologically active salivary gland molecules is presented in a recent review [28].

### **P672 and CCL8**

P672 is a chemokine binding protein (evasin). Evasins bind to multiple chemokines of different origin and their effects are thus pleiotropic. To date, several evasins originating in tick saliva have been identified [160, 161] and they inhibit responses of many chemokine sensitive molecules including neutrophiles or macrophages, which have been demonstrated in several tick species [28]. P672 was originally identified in *Rhipicephalus pulchellus* and its promiscuous binding abilities assign it 13 different chemokine partners showing different dissociation constants. Mass spectrometric characterisation revealed the presence of several *N-*linked glycans and their deprival negatively influences the affinity of P672 to CCL8, although the underlying mechanism of this observation is yet to be uncovered [162].

### **Protease inhibitors**

Many of the tick salivary proteins are glycosylated [163]. While the exact structure of the glycans attached to these proteins has not been studied, research has concentrated on the role of glycosylation with regard to the recognition of glycans by host immune systems. The importance of the glycan part for antibody recognition was shown for several proteins, such as AamS6 serpin [164], *R. microplus* serpins [165] or evasins 1 and 3 [166] confirming the need to use of glycosylated recombinant proteins in anti-tick vaccine preparations.

For proteins, where the role of glycosylation for the protein function was not confirmed, masking of the tick proteins antigenic epitopes and thus minimization of the immune response was speculated as the role of glycosylation [166].

#### ***Serpin 19***

Serpin 19 is a serine protease inhibitor identified in the saliva of *Amblyomma americanum*. Serpin 19 displays a broad range of inhibitory activities: it interferes with the host homeostasis, coagulation and the development of inflammatory response. Importantly, the activity of many serine proteases is both positively and negatively regulated when bound to GAGs [167–169] and serpin 19 also contains several predicted GAG binding motives [170]. The functional validation further confirmed its GAG-binding properties and also extended the list of binding partners with heparin sulphate and heparin [170].

#### ***Variegin***

The inhibition of blood coagulation cascade represents an important property of tick saliva that facilitates successful engorgement on the host. Variegin is a small thrombin-binding oligopeptide isolated from *A. variegatum* salivary glands. During tick feeding, variegin binds thrombin and disables its fibrinolytic activity and thus blocks the blood coagulation cascade. Despite its small size, variegin possesses a single *O*-linked glycan [171]. The synthetic *O*-glycosylated variegin analogues show significantly higher affinity to thrombin and consequently lower reaction kinetics of thrombin-mediated fibrinogenolysis compared to the non-glycosylated form, confirming the importance of its glycosylation. The functional analysis of the inhibition mechanism using macromolecular docking revealed the formation of some favourable hydrogen bonds between hydroxyl groups of the glycan and the allosterically important sites of thrombin [172].

## **Glycosylation in the*Anaplasma*infection cycle**

*Anaplasma* is a genus of gram-negative rickettsial bacteria. They are obligate intracellular parasites infecting mammals including many domestic animals. The infection causes a reduction of the animal’s body weight, abortions, reduces milk production and frequently leads to death [173–175]. In humans, *A. phagocytophilum* is the only confirmed pathogenic species causing human granulocytic anaplasmosis. Patients suffer from fever, headache, myalgias, chills, leukopenia, thrombocytopenia and liver damage manifested by elevated liver enzymes in serum [176]. The symptoms are usually mild but for some individuals, e.g. patients with a weakened immune system, it can be fatal. The infected vertebrate host serves as a reservoir where the bacterium can proliferate for many years and infect naïve ticks [177].

The main vectors of the genus *Anaplasma* are ticks, especially species of the genera *Ixodes*, *Dermacentor*, *Rhipicephalus* and *Amblyomma* [178, 179]. The initial phase of the infection during colonization of the host is the recognition of a suitable cell, attachment onto this cell, and entry into it. This process is facilitated by several specialized bacterial proteins (adhesins/invasins) that can recognize host surface molecules including glycans and glycoproteins and initiate signalling cascades to promote pathogen internalization. *Anaplasma* spp. express several surface proteins which are involved in binding to glycosylated host cells receptors and thus in the infection of the host and tick cells. These differ in glycan specificity and importance for the infection of various hosts and host cell types.

### ***Anaplasma*glycoprotein-binding surface proteins**

As an intracellular pathogen, *Anaplasma* depends on a host cell to survive. *Anaplasma* infects two different types (groups) of organisms: the tick vector and the mammalian hosts, with various cell types being infected by the pathogen. Recognition of the cell type and of the infected organism is provided through binding of surface glycan epitopes or even several epitopes on a glycoprotein molecules.

Two groups of *Anaplasma* surface proteins were shown to recognize tick or host glycoproteins: outer membrane proteins (Omps) and major surface proteins (MSPs).

*OmpA* belongs to highly conserved genes among *A. phagocytophilum* isolates and is transcriptionally induced during feeding of *A. phagocytophilum*-infected ticks on mice and also during the invasion of mammalian but not tick cells [180, 181]. Pre-treatment of *A. phagocytophilum* or *A. marginale* bacteria with the respective OmpA antiserum reduces their ability to infect mammalian cells [181, 182]. Also, preincubation of mammalian cells with a recombinant ApOmpA effectively inhibits *A. phagocytophilum* infection of host cells.

Glycoproteins containing α1,3-fucose and either sLex or 6-sulfo sLex on host cells are recognized by the outer membrane protein A (ApOmpA) of *A. phagocytophilum* [180, 181]. On the other hand, OmpA of *A. marginale* (AmOmpA), a species non-pathogenic for humans, binds only α1,3-fucose and sLex but not 6-sulfo-sLex glycans. *Anaplasma marginale* also produces AmOmpA in both the infected mammalian and tick cells. Pre-treatment of host cells with sialidase or trypsin reduces or nearly eliminates OmpA adhesion. Therefore, AmOmpA interacts with sialylated glycoproteins *via* an adhesin-receptor pair. Thus, both AmOmpA and ApOmpA recognize different receptor molecules even though these receptors share some structural similarity and thus provide a similar function to these two bacterial species [182].

Structures of *A. marginale* and *A. phagocytophilum* OmpA proteins are very similar and their binding domains are structurally conserved. The OmpA binding domain was identified within amino acids 59 to 74 and it is responsible for the recognition of α2,3-bound sialic acid and α1,3-fucose [180]. A recent study by Hebert et al. [182] describes the OmpA receptor-binding domain between the amino acids 19 to 74.

Another group of surface proteins interacting with host (glycosylated) molecules are the major surface proteins (MSPs) that are involved in the adhesion of host cells and the immunological reaction of the host [183–187]. MSP1 protein with its variants α, β1, and β2 and the MSP3 protein are present in *A. marginale*, while MSP2 and MSP4 in both *A*. *marginale* and *A. phagocytophilum* [188]. The MSP1 complex consists of two polypeptides MSP1a and MSP1b and both polypeptides participate in adhesion processes to both tick cells and bovine erythrocytes [183–186].

Similarly to OmpA, these proteins show glycan-binding activity. *Anaplasma marginale* MSP1 and MSP2 can hemagglutinate bovine erythrocytes [184] suggesting recognition of some erythrocyte surface saccharide molecules. Recombinant forms of the MSP1 isoforms are glycosylated; MSP1a recombinant glycoprotein contains glucose, galactose, mannose and xylose, while MSP1b contains glucose, galactose and mannose. The functional domain of MSP1a contains tandemly repeated peptides that are important for adhesion to tick cells and bovine erythrocytes. The MSP1a polypeptide backbone alone shows binding to tick cell extract proteins and the glycan in its N-terminus enhances this binding [189, 190]. The MSP2 protein binds to the mammalian PSGL-1 [191] and thus can be responsible for the above mentioned *Anaplasma* recognition of sialic acid on this protein. A hypervariable region is present in the middle of the MSP2 gene which allows the bacterium to express various paralogs of the protein on its surface, possibly enhancing immune system evasion [192, 193]. However, the glycan binding abilities of the various MSP2 paralogs were not studied.

In addition to the above-described receptor molecules, two other proteins, Asp14 and AipA, were found to be acting together with OmpA during the infection of host cells. However, neither of these two proteins were shown to bind glycans nor to be glycosylated [180]. Finally, during the past ten years, other novel *A. phagocytophilum* surface proteins Asp55, Asp62 and APH_1235, with possible function as adhesins and invasins have been identified [194–196]; however, their receptor molecules remain unknown.

### ***Anaplasma*-host interactions**

A confirmation of *Anaplasma* recognition of host-surface glycans came by Goodman et al. [197] showing binding of *A. phagocytophilum* to the cell surface of the promyelocytic leukaemia cell line HL-60. Bacterial binding to the cell surface correlates with the expression of the sialyl Lewis x (sLex) or a closely related 6-sulpho sLex glycan-containing molecules glycan and α1,3-fucosylated molecules. On the other hand, α1,3-fucosylated glycans but not sialylated glycans, are essential for the infection of murine and human mast cells by *A. phagocytophilum* [180, 198]. These glycan epitopes are important for *Anaplasma in vivo* as has been shown by Carlyon et al. [199].

The protein part bearing the recognized glycans can be also important; thus, not any glycan molecule is recognized, only the one found on a specific protein. In humans and animal hosts, *A. phagocytophilum* exhibits, amongst others, a tropism for myeloid cells. As an adhesion molecule involved in the binding to the surface of human neutrophils, the P-selectin glycoprotein ligand-1, PSGL-1, has been identified [197, 199–202]. In the case of human PSGL-1, *A. phagocytophilum* cooperatively binds to a short amino acid sequence in its *N*-terminal region and an *O*-glycan containing a sialyl Lewis x (sLex) on PSGL-1 (NeuAcα2,3Galβ1,4[Fucα1,3]GlcNac) [202] or on another molecule. On the other hand, PSGL-1 is not the major ligand in mice [199, 200]. Thus, the terminal or core α1,3-fucosylated glycans seem to be a generally recognized receptor, while sialylated glycans and PSGL-1 enhance the infection of diverse types of mammalian host cells.

### ***Anaplasma*-vector interactions**

In the pathogen-tick relationship, several tick glycosylated molecules can be induced in the presence of a pathogen in the tick tissues and help the pathogen to colonize the tick or enhance its infection. For example, α1,3-core-fucosylated glycans are required for tick colonization by *A. phagocytophilum* and silencing of the responsible fucosyltransferases results in the absence of *Anaplasma* in the infected ticks. To increase the number of its receptors in the tick, *A. phagocytophilum* induces the expression of α1,3-fucosyltransferases to enhance the colonization of *I. scapularis* ticks. Therefore, α 1,3-fucose is a unifying determinant that *A. phagocytophilum* targets to infect its natural murine and arthropod reservoirs and accidental human hosts as well. In addition, the presence or absence of these glycans does not affect the transmission of the pathogen from the tick vector to the vertebrate host. While the infection of the tick by *Anaplasma* depends on the presence of α1,3-core-fucosylated glycans, these epitopes do not seem to be important for the infection by another tick-borne pathogen, *B. burgdorferi* [203].

Furthermore, tandem repeat peptides of the MSP1a functional domain are important for the adhesion of bacteria to tick cells and the glycosylation of MSP1a probably plays a role during the adhesion of *A. marginale* to tick cells [189, 190].

Colonization of the tick by pathogens depends on the tick life-cycle; one of the crucial steps is the colonization of the midgut or survival in the midgut in the process of the blood meal digestion. For successful colonization, the tick midgut peritrophic matrix (PM) and bacterial biofilms formed in the midgut are critical. The PM forms a barrier between the midgut lumen and the epithelial cells lining the luminal side of the midgut and is formed by a thick matrix of mostly chitin with various proteins, such as chitin deacetylase, and glycoproteins [204]. One of the bacteria depending on the biofilm formation in the *I. scapularis* tick midgut is *A. phagocytophilum*. The presence of this bacterium affects the midgut microbial community and biofilm composition and it also decreases the expression of several genes for the glycoprotein peritrophin, one of the major PM components. This results also in decreased PM thickness. Furthermore, RNAi silencing of these genes significantly enhanced *Anaplasma* colonization of the tick [205]. *Anaplasma* further enhances its chances for a successful colonization of *I. scapularis* ticks by induction of an antifreeze protein (IAFGP) during the infection of ticks [205]. This secreted antifreeze glycoprotein inhibits bacterial biofilm formation through binding to the D-alanine residue of some bacteria peptidoglycan and was induced in response to *Anaplasma* infection [206, 207]. IAFGP expression resulted in thinning of the tick midgut PM and RNAi silencing of *iafgp* gene resulted in the absence of *Anaplasma* in the tick midgut [205].

## **Tick lectins**

Ticks, like other arthropods, lack specific adaptive immunity. To defend themselves against invading microorganisms, ticks use the evolutionarily older nonspecific innate immune system, including both cellular and humoral immune responses. Cellular immune reactions involve haemocytes capable of phagocytosis, encapsulation or nodulation of foreign microorganisms and particles. The humoral immune response involves a range of non-specific pathogen-recognizing defence systems: PRRs, lectins, complement-like molecules, pro-phenoloxidase activation, haemolymph coagulation factors, antimicrobial peptides, reactive oxygen species, etc. Some of these molecules which function as mediators in the innate immune response are glycosylated and/or may recognize glycan-containing epitopes, e.g. recognition receptors for pathogens, complement-related molecules, or lectins (Table 3). In mammals, lectins play an important role in the recognition of specific glycosylated surface molecules of a variety of pathogens (PAMPs) and subsequent activation of the lectin pathway [208, 209]. MBL or ficolins known to recognize *N*-acetyl groups [210] serve as the recognition molecules, which are further integrated with the MBL-associated serine proteases to trigger the complement activation.

**Table 3:** Overview of identified tick lectins. Lectins identified in different tick species are listed including the tissue where the lectin was identified. Lectin binding specificity, its function and molecular weight are also listed if known

Lectin	Species	Tick tissue	Specificity	MW (kDa)	Function	Reference
Galectins (OmGalec)	*O. moubata*	Haemocytes, midgut, SG, ovaries	Lactosamine-like disaccharides	37.4	Putative functions in tick development, immunity, and vector-pathogen interaction	[221]
Dorin M	*O. moubata*	Haemocytes	*N*-acetyl-D-hexosamines and Sialic acid specific	na	Pattern recognition molecules	[214]
OMFREP	*O. moubata*	Hemolymph, salivary glands	Probably similar to Dorin M	na	Probably similar to Dorin M	[215]
Ixoderin A	*I. ricinus*	Hemolymph, salivary glands, midgut	Peptidoglycan recognition protein?	na	Putative defence protein, identification of self-/non-self tissues	[215, 219]
Ixoderin B	*I. ricinus*	Salivary glands	Unknown	na	Unknown putative immunomodulatory function	[215, 219]
Hemelipoglycoprotein	*D. marginatus*	Haemocytes, salivary glands, gut	Galactose- and mannose-binding specificity	290, 2 subunits	Putative innate immunity	[220]
Unknown lectin	*I. ricinus*	Gut, hemolymph	Sialic acid, *N*-acetyl-glucosamine	85	Putative recognition molecule	[233]
Unknown lectin	*I. ricinus*	SGs	Sialic acid	70	Unknown	[233]
TSLPI	*I. scapularis*	Unknown	Mannan	na	Unknown	[42]
HICLec	*H. longicornis*	Midgut, ovary	Unknown	60.2	Unknown	[223]
Serpin 19	*A. americanum*	Saliva	GAGs	43.0	Serine protease inhibitor	[170]

*Abbreviations*: *MW* molecular weight, *na* not available

### **Fibrinogen-related proteins**

Invertebrates contain a variety of fibrinogen-related proteins (FRePs), all of them sharing structural similarity with fibrinogen. A common feature of FRePs is their glycan-binding activity as they recognize the invading pathogen through its specific glycan epitopes. Their expression increases upon infection of the invertebrate by parasites or by pathogens [211, 212] with possibly a specific role in complement activation [213]. However, some of the tick FRePs family proteins (such as ixoderins described below) may have various other functions (Table 3).

Dorin M from the soft tick *Ornithodoros moubata*, the first lectin purified and characterized from any tick species, shows a strong similarity to ficolins but lacks the N-terminal collagen domain [214, 215]. Dorin M and its closest homologue OMFREP, also from *O. moubata*, share sequence similarity with the innate immune FRePs Tachylectin 5A and B from the horseshoe crab, *Tachypleus tridentatus* [215, 216]. It has a binding activity for sialic acid [214], its conjugates and *N*-acetyl-hexosamines. The protein has three *N*-glycosylation sites modified by high-mannose type glycans and core-fucosylated paucimannose glycans [217]. Other FRePs were later identified in the haemolymph of *D. marginatus*, *R. appendiculatus*, *R. pulchellus* and *R. sanguineus* based on the cross-reactivity with sera directed against Dorin M [218].

The hard tick *I. ricinus* contains several FReP encoding sequences in its genome (ixoderins A, B and C) and their analogues are present in *I. scapularis* as well. While proteins similar to ixoderins A and C are present also in other tick species, ixoderin B-like proteins are found only in the genus *Ixodes*. All these proteins contain predicted glycosylation sites and they contain the fibrinogen-like domain with carbohydrate-binding properties [213, 215]. In *I. ricinus*, the expression of ixoderin A is restricted to haemocytes, salivary glands, and midgut while ixoderin B is only expressed in salivary glands [215]. As expected based on published information on other invertebrate FRePs, ixoderins are also involved in defence against pathogens. Namely, ixoderins A and B are involved in phagocytosis of some pathogens as shown for *Candida albicans* [219]. On the other hand, knockdown of these two ixoderins did not affect the phagocytosis of the tick-transmitted *B. afzelii* and knockdown of all three ixoderins does not affect its transmission [219]. The reason can be the missing protein glycosylation and thus the binding site for these lectins on the *Borrelia* surface [37]. Ixoderins and FRePs can be involved in other processes as well; ixoderin B may be involved in the matrix attachment processes and angiogenesis inhibition. Alternatively, it may antagonize the effect of host ficolin [215].

Finally, one of the tick storage proteins, hemelipoglycoprotein, from several hard tick species seems to share a structural similarity to FRePs with its primary sequence showing a high similarity to the fibrinogen domain [218, 220].

### **Other tick lectins**

OmGalec from the soft tick *O. moubata* is the first member of galectin family identified in ticks with the specificity towards β1-3 and β1-4 bound galactose to GlcNAc, and Glc and α1-3 bound galactose to GalNAc [221]. Similar proteins are also present in *R. appendiculatus* and *I. scapularis* [188]. OmGalec contains two carbohydrate-binding domains which share low sequence similarity and thus possibly possesses a different saccharide-specificity. The protein is expressed in various life-stages and tissues, with the highest expression in haemocytes, midguts and ovaries [221]. It has been shown that galectins play a vital role in immune homeostasis by being pathogen recognition receptors [222].

C-type lectins are also present in the available tick genomes and transcriptomes [223, 224]. The only characterized C-type lectin from *Haemaphysalis longicornis* (HlCLec) contains three various carbohydrate-binding domains. Each of them has been shown to recognize the bacteria *E. coli* and *S. aureus* and participate in the tick defence against gram-negative bacteria, but they do not have a direct effect on bacterial growth. HlCLec also affects the blood-feeding process and affects larvae hatching and mortality. Expression of this lectin is increased during blood-feeding and is the highest in the midgut and ovary [223]. In mosquitoes, C-type lectins influence the midgut colonization by bacteria midgut microbiome [225] and facilitate infection with West Nile and dengue viruses [226, 227].

Calreticulin (CRT), a lectin chaperone responsible mainly for the control and proper folding of glycoproteins, is conserved in all tick species and is even used as the biomarker for human tick bites in *I. scapularis* [228]. In blood-feeding parasites, CRTs participate in evasion of the host defence mechanisms, namely the complement by binding the initiator of this pathway, the C1q protein, or factor Xa participating in the blood coagulation [229]. In mammals, CRT on the surface of neutrophils also binds C1q as well as other immune-related lectins [230]. Similarly, the salivary secreted CRT from *A. americanum* binds host C1q. On the other hand, it does not bind the factor Xa and does not inhibit the activation of the classical complement cascade and host haemostasis. The *A. americanum* CRT shares a very high sequence similarity with other tick CRTs and thus similar functions of tick CRT can be expected [231].

Several other lectins are characterized in *I. ricinus*, but have not been identified to date: the 37, 60, 65, and 73 kDa lectins from midgut showing haemagglutination activity [163, 232]. The 37 kDa lectin has a binding specificity towards β1-3 glucan, while the 65 kDa protein binds bovine submaxillary mucin, containing a complicated mixture of various glycan structures and more specifically binds free sialic acid. Another lectin is present in haemolymph/haemocytes with a molecular weight of 85 kDa. It is a C-type lectin with specificity towards sialic acid and GlcNAc [233]. Several other lectins with haemagglutination activity have also been described in other ticks including *R. appendiculatus* [234, 235], *O. tartakovskyi*, *O. tholozani* and *A. polonicus* [233].

## **Tick glycans**

**Fig. 2:**
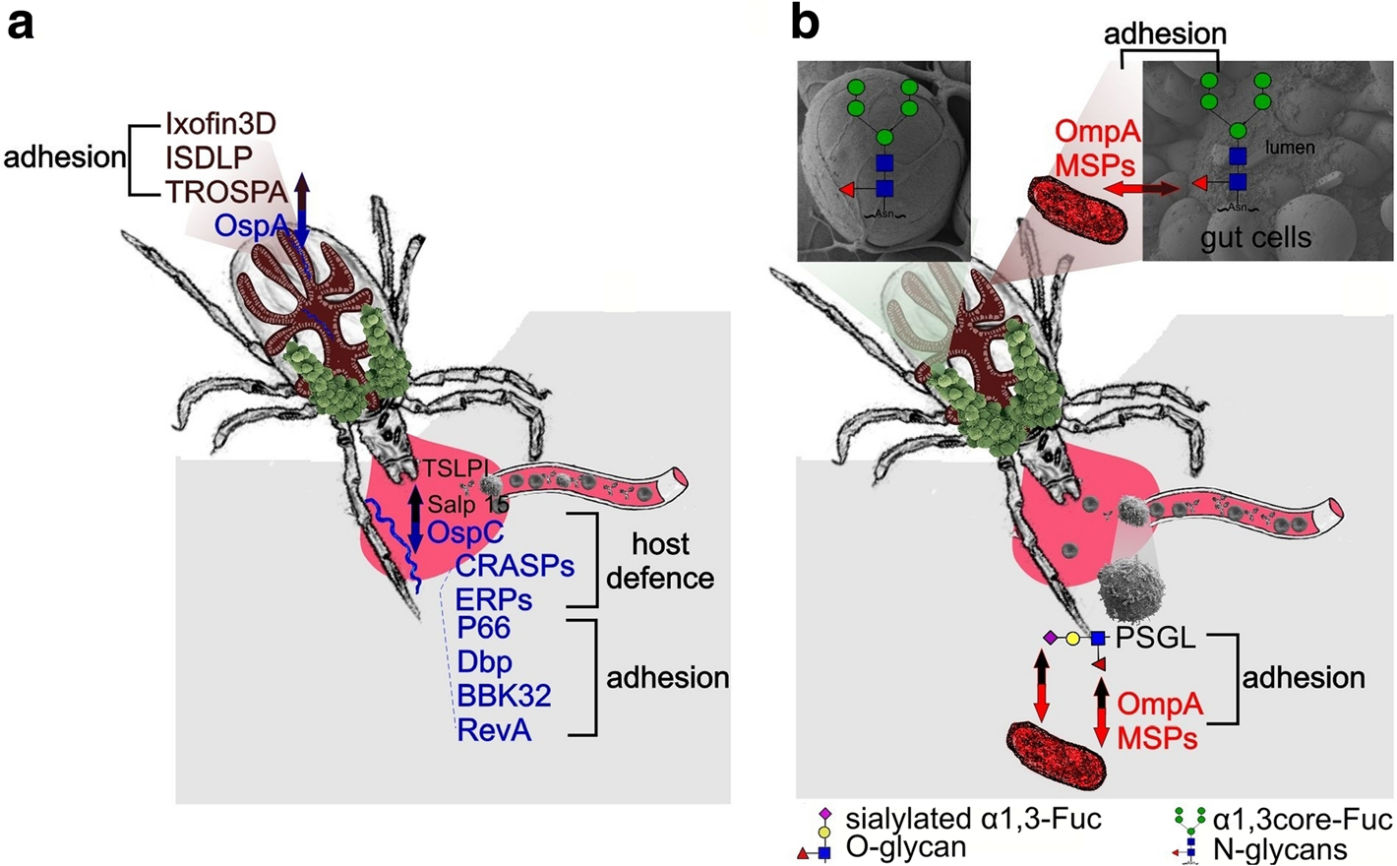
Scheme of a model complex-type glycan showing presented oligosaccharide structures. An example of an *N*-glycan bearing the three glycoepitopes immunogenic in the mammalian hosts are shown. The αGal epitope is formed by a terminal galactose bound to another galactose *via* an α1-3 bond. In the case of the core α1,3-fucose, both the specific α1,3 bond and the core (not terminal localization of fucose are important for the immunogenicity in mammals. Two types of sialic acid are present in Eukaryotes: the *N*-acetylneuraminic acid (Neu5Ac) and the *N*-glycolylneuraminic acid (Neu5Gc). As humans do not possess the enzymatic apparatus for the production of Neu5Gc into glycans, glycans terminated with this type of sialic acid are immunogenic in humans. The used symbol nomenclature is based on the Consortium for Functional Glycomics (http://www.functionalglycomics.org/)

Regarding the glycans and glycoproteins of blood-feeding arthropods, several studies describe these molecules using lectin staining and other indirect methods. Lectin studies show the presence of both *N-* and *O-*glycosylated proteins in tick tissues and some glycoproteins have been shown to be antigenic determinants for the immune response of the host [236–239]. In recent years, the direct determination of glycan structures and composition, mostly using mass spectrometry, has also been published, either from tick tissues and cells [203, 240] or purified proteins [220]. The three most interesting glycan structures related to host-parasite interaction and host immune system reaction are described below; representation of these structures in a glycan molecule is shown in Fig. 2. An overview of tick glycans with known structures is listed in Table 4.

**Table 4:** Overview of identified tick glycan structures composition. Monosaccharide compositions of the identified N-glycans are shown. Note that in some cases, the same composition can define various structures. For each glycan, the protein or the tick samples is listed, in which it was identified by mass spectrometry

Glycan composition				Protein/sample	Reference
Paucimannose glycans					
HexNAc	Hex	dHex	Sia		
2	3	0	0	Dorin M (position ^41^NHS, ^171^NGS, ^129^NHS) from *O. moubata*	[217]
				*I. ricinus* fed female salivary glands	[240]
				*I. ricinus* fed female midgut	[240]
				*I. scapularis* salivary gland	[203]
2	4	0	0	Dorin M (position ^41^NHS, ^171^NGS, ^129^NHS) from *O. moubata*	[217]
				*I. ricinus* fed female salivary glands	[240]
				*I. ricinus* fed female midgut	[240]
				*I. scapularis* salivary gland	[203]
High-mannose glycans					
HexNAc	Hex	dHex	Sia		
2	5	0	0	Dorin M (position ^41^NHS, ^129^NHS) from *O. moubata*	[217]
				*I. ricinus* fed female salivary glands	[240]
				*I. ricinus* fed female midgut	[240]
				*I. scapularis* salivary gland	[203]
2	6	0	0	Dorin M (position ^41^NHS, ^129^NHS) from *O. moubata*	[217]
				*I. ricinus* fed female salivary glands	[240]
				*I. ricinus* fed female midgut	[240]
				*I. scapularis* salivary gland	[203]
2	7	0	0	Dorin M (position ^41^NHS, ^129^NHS) from *O. moubata*	[217]
				*I. ricinus* fed female midgut	[240]
				*I. scapularis* salivary gland	[203]
2	8	0	0	Dorin M (position ^41^NHS, ^129^NHS) from *O. moubata*	[217]
				*I. ricinus* fed female midgut	[240]
				*I. scapularis* salivary gland	[203]
2	9	0	0	Dorin M (position ^41^NHS, ^129^NHS) from *O. moubata*	[217]
				*I. ricinus* fed female salivary glands	[240]
				*I. ricinus* fed female midgut	[240]
				*D. marginatus* Hemelipoglycoprotein	[220]
				*I. scapularis* salivary gland	[203]
2	10	0	0	*D. marginatus* Hemelipoglycoprotein	[220]
				*I. scapularis* salivary gland	[203]
Core-fucosylated glycans					
HexNAc	Hex	dHex	Sia		
2	3	1	0	Dorin M (position ^171^NGS) from *O. moubata*	[217]
				*I. ricinus* fed female salivary glands	[240]
				*I. ricinus* fed female midgut	[240]
				*I. scapularis* salivary gland	[203]
2	4	1	0	Dorin M (position ^171^NGS) from *O. moubata*	[217]
				*I. ricinus* fed female salivary glands	[240]
				*I. ricinus* fed female midgut	[240]
				*I. scapularis* salivary gland	[203]
2	5	1	0	Dorin M (position ^171^NGS) from *O. moubata*	[217]
				*I. ricinus* fed female salivary glands	[240]
				*I. ricinus* fed female midgut	[240]
				*I. scapularis* salivary gland	[203]
3	2	1	0	*I. ricinus* fed female salivary glands	[240]
3	3	1	0	*I. ricinus* fed female salivary glands	[240]
				*I. ricinus* fed female midgut	[240]
				*I. scapularis* salivary gland	[203]
4	3	1	0	*I. ricinus* fed female midgut	[240]
				*I. scapularis* salivary gland	[203]
4	4	1	0	*I. scapularis* salivary gland	[203]
4	5	1	0	*I. ricinus* fed female midgut	[240]
				*I. scapularis* salivary gland	[203]
4	6	1	0	*I. ricinus* fed female midgut	[240]
5	3	1	0	*I. ricinus* fed female salivary glands	[240]
				*I. scapularis* salivary gland	[203]
5	5	1	0	*I. ricinus* fed female midgut	[240]
6	6	1	0	*I. ricinus* fed female midgut	[240]
Complex glycans					
HexNAc	Hex	dHex	Sia		
3	4	0	0	*I. ricinus* fed female salivary glands	[240]
4	5	0	0	*I. ricinus* fed female salivary glands	[240]
				*I. ricinus* fed female midgut	[240]
4	6	0	0	*I. ricinus* fed female midgut	[240]
4	7	0	0	*D. marginatus* Hemelipoglycoprotein	[220]
4	8	0	0	*D. marginatus* Hemelipoglycoprotein	[220]
5	6	0	0	*I. ricinus* fed female midgut	[240]
6	2	0	0	*I. ricinus* fed female midgut	[240]
Sialylated glycans (containing either Neu5Ac or Neu5Gc)					
HexNAc	Hex	dHex	Sia		
4	5	0	1	*I. ricinus* fed female salivary glands	[240]
				*I. ricinus* fed female midgut	[240]
4	5	0	2	*I. ricinus* fed female midgut	[240]
5	6	0	1	*I. ricinus* fed female midgut	[240]
5	6	0	2	*I. ricinus* fed female midgut	[240]

*Abbreviations*: *HexNAc N*-acetyl-hexosamine (*N*-acetyl-glucosamine or *N*-acetyl-galactosamine), *Hex* hexose (mannose, glucose, galactose), *dHex* deoxyhexose (fucose), *Sia* sialic acid (*N*-acetyl-neuraminic acid, *N*-glycolyl-neuraminic acid)

### **Alpha-galactose epitope**

Alpha-galactose epitopes (Galα1-3Gal; αGal) are abundant on glycolipids and glycoproteins of plants, arthropods and non-primate mammals [241]. αGal is a novel allergen identified first during clinical trials in 2004 in patients treated with cetuximab, a medical preparation for metastatic colorectal cancer treatment. Several cases of hypersensitivity reaction were registered soon after cetuximab administration into the blood due to the presence of αGal in its structure. The majority of sensitive individuals come from a population in south-eastern USA [242]. Furthermore, the geographical distribution of cases with cetuximab hypersensitivity corresponded to the distribution of red meat allergy cases and tick prevalence. Additionally, patients with red meat allergy experienced a tick bite in the months preceding the allergy symptoms. The causative agent of the αGal sensitization in the south-eastern region of the USA is the lone star tick *A. americanum* [243]. Red meat allergy is also linked with *I. holocyclus* tick bite in the Australian population [244] . Conversely, a bite by the *I. scapularis* tick from the same genus in the USA does not seem to result in red meat allergy [243]. Lastly, Chinuki et al. [245] described the allergy development upon *H. longicornis* bite in Japan. Direct evidence on αGal epitopes presence in *I. ricinus* is provided by Hamsten et al. [246], specifically in the tick midgut. However, the presence of αGal just in the tick saliva is what is important for patient sensitization. In this regard, the presence of undigested complete host proteins and glycoproteins was described in the tick body and, importantly, in the tick saliva [240, 246, 247] and thus the presence of αGal originating in the blood of non-mammalian hosts from the previous blood-feeding can be expected in the saliva. The αGal epitope is only known to be present in the saliva of *A. sculptum*, a tick that until now has not been connected with red meat allergy cases [248].

### **Core α1,3-fucosylation**

The allergenic core α1,3-fucose (α1,3-Fuc) attached on the proximal GlcNAc residue is widely present in plants and arthropods and is one of the well-known possible human allergens as it is usually absent in mammals. It can induce production of specific IgE antibodies associated with IgE-mediated allergic immune responses, which is mostly described for schistosomes or venoms of some species of the order Hymenoptera. However, such a response is not described after a tick bite [249–251]. It is rather surprising, as α1,3-fucosylated structures are present in the tick salivary glands as well as in saliva of both *I. ricinus* and *I. scapularis* [203, 252]. This can be explained by the structural features of the allergenic epitopes; for example, in the case of core α1,3-Fuc, terminal GlcNAc weakens the immune response [253]. Additionally, more than one epitope has to be present to trigger the allergic reaction and the presence of blocking IgG4 antibodies against this epitope can lower the immune reaction [249].

The α1,3-Fuc modification of the *N-*linked glycan core mediates an entrance of one of the tick-transmitted pathogens, *A. phagocytophilum*, into *I. scapularis* midgut cells, but it is not required for the transmission of the pathogen to a vertebrate host. Furthermore, *Anaplasma* increases the expression of α1,3-fucosyltransferases in the tick, further increasing its ability to infect the tick. On the other hand, the infection of the tick by *B. burgdorferi* was not affected by the presence or absence of core α1,3-Fuc [203].

### **Sialic acids**

Sialic acids (Sia) are found typically in the terminal position of vertebrate complex *N-* or *O-*linked glycans. In insects, some studies have shown the ability of sialylation [254, 255] and the importance of sialylation for insectdevelopment, even though the abundance of sialylated glycans is very low [256].

*N*-glycans terminated with Sia are present also in the organs of the tick *I. ricinus*, namely in the gut, salivary glands, ovary and Malpighian tubules [240, 257]. However, the sialylated proteins in the adult ticks originate most probably from the host blood [258]. Hypothetically, sialoglycans present in the tick organs and in the secreted tick saliva can be engaged in molecular mimicry. We suppose that sialic acid is produced also by the tick itself in the ovary and eggs and later in larvae; the exact role of the tick sialylated proteins for the physiology and development of ticks is not yet known (unpublished results). Both eukaryotic types of sialic acids, *N*-acetyl-neuraminic acid and *N*-glycolyl-neuraminic acid (Fig. 2), were detected in the ticks [240].

## ***N*-linked glycans of flaviviruses**

Tick-borne encephalitis virus (TBEV), a member of the genus *Flavivirus*, can cause serious infections in humans, which may result in encephalitis/meningoencephalitis. The viral single-stranded genomic RNA of positive polarity contains one open reading frame, which encodes a single polyprotein that is co-translationally and post-translationally cleaved by viral and cellular proteases into three structural and seven non-structural proteins (Table 4) [259].

### **Flaviviral non-structural proteins**

Non-structural proteins of the flavivirus family (NS1, NS2A, NS2B, NS3, NS4A, NS4B and NS5) do not have their precise role elucidated, but they are generally considered as the effectors of viral replication, which occurs in close association with cellular membranes. Dramatic changes in the intracellular membrane structures including convoluted membranes, vesicle packets or paracrystalline arrays were observed as a result of dengue virus (DENV), TBEV or West Nile virus (WNV) replication [260–262]. Recently, NS1, NS4A and possibly also NS2A, were described to be involved in the formation of vesicle packets [263, 264]. Moreover, novel functions in terms of virus-host interactions were recently described for particular NS proteins; for example, TBEV NS5 protein acts as an inhibitor of interferon-activated Jak-STAT signalling [265].

### **Flaviviral structural proteins**

Apart from seven non-structural proteins, the flaviviral genome encodes three structural proteins (C, M, E). The flaviviral nucleocapsid is composed of (+) ssRNA genome and the capsid protein (C), whereas the host-derived envelope contains two glycoproteins, the membrane (prM/M) protein and the envelope (E) protein [266].

The E glycoprotein is localized in the viral envelope and is the main antigenic determinant of TBEV inducing a humoral immune response. It mediates fusion of TBEV with host cell membrane and thus facilitates the virus entry to the host cell. It forms heterodimers with the prM protein; the prM-E protein interaction is essential for proper folding of E protein [267]. The heterodimers then migrate to the ER membrane and eventually bud off as nucleocapsid-containing immature virions [260, 268].

The M glycoprotein is an integral part of the viral envelope together with E protein. It forms heterodimers with the E protein and functions as a chaperon ensuring proper folding of E protein [267]. Non-infectious immature virions containing prM/E homodimers undergo maturation process in late trans-Golgi network by cleavage of pr part from the prM protein by host protease furin. The cleavage produces protein M and triggers re-organization of protein E to form homodimers [268, 269].

### **TBEV proteins glycosylation**

The glycosylation of viral proteins increases their folding efficiency and promotes their intracellular transport by the interaction with host lectins [270]. In several viruses that cross the endoplasmic reticulum during their life-cycle, the protein glycosylation was proven important for virus growth, budding, secretion, and pathogenicity (reviewed in [271–273]). So far, only membrane glycoproteins prM/M and E are known to be *N*-glycosylated in TBEV (Table 5) [274–276]. α1,3-core fucosylated, high-mannose and hybrid N-glycans were shown to be present in the E protein of TBEV produced in chicken embryos by affinoblots [277]. E protein glycosylation was also confirmed recently also using cryo-electron microscopy, even though the exact glycan structure was not defined [278]. Another two sites (N130 and N207) in the NS1 protein are *N*-glycosylated in the case of dengue virus [274, 279]. One of these two *N*-glycosylation sites (N207) is present also in the TBEV NS1 protein; however, its glycosylation has not yet been shown (Table 5).

**Table 5:** TBEV protein glycosylation overview. List of TBEV proteins and their functions. Identified or predicted N-linked glycosylation sites are listed as well. NetNGlyc 1.0 Server was used for N-linked glycosylation site prediction

	Protein	Function	*N*-linked glycosylation	Reference
Structural	C	Capsid protein; forming of nucleocapsid	None	
	prM/M	Envelope protein; E protein chaperone	N32	[265, 302]
	E	Envelope protein; binding and fusion	N154, N361	[263–265]
Non-structural	NS1	Replication	Predicted: N85, N207	
	NS2A	Assembly, replication	None	
	NS2B	NS3 serine-protease cofactor	None	
	NS3	Serine-protease, helicase, replication RNA triphosphatase	Predicted: N160, N499, N555	
	NS4A	Assembly, replication	None	
	NS4B	Assembly, induction of membrane rearrangements	Predicted: N188	
	NS5	Methyltransferase, RNA-dependent RNA polymerase	Predicted: N18, N175, N215	

The E glycoprotein is a viral surface protein and thus contains major antigenic epitopes responsible for triggering the host immune system [280]. The *N*-glycosylation site at position N154 is present in the majority of TBEV strains and other flaviviruses. Moreover, a potential N361 glycosylation site is present in TBEV E protein as well. Depending on the strain, zero to two glycans are attached [280, 281]. The investigation of the presence and position of E protein glycans showed that an increased number of E protein glycans elevate its expression. Conversely, the E protein glycosylation deletion mutants showed reduced E protein production, suggesting the importance of glycosylation for the viral life-cycle [276].

Importantly, the absence of E-protein glycosylation affects the E protein conformation and further the TBEV infectivity only in the mammalian host, but not in the tick vector. Different temperatures in the host (37 °C) and the vector (23 °C) do not affect the stability of the deglycosylated E protein [282]. In light of this evidence, the E protein glycosylation seems to represent one of the factors conferring different vector and host competence. Interestingly, the investigation of mosquito-borne flaviviruses, DENV and WNV, shows that glycans modifying E protein are important for virus propagation in both vector and host cells. However, the role of the particular *N*-linked glycosylation site varies depending on the invertebrate/vertebrate host [283–285]. For example, the importance of *N*-linked glycan at 154 aa of WNV E protein was proved in case of the vector (*Culex pipiens* and *Cx. tarsalis* mosquitoes) as well as the bird host (*Gallus gallus*) [286, 287].

The prM protein encodes for the precursor of membrane protein M and also contains one *N*-linked glycosylation consensus sequence in N32 position (Table 5) [267, 276]. During TBEV maturation, the structural proteins prM and E form heterodimer, where prM has a chaperone-like role in the folding and maturation of E protein [267], although the biological role of TBEV prM glycosylation, has not yet been elucidated. However, Goto et al. [279] suggest the participation of carbohydrate-mediated interaction for prM-E heterodimer formation; glycosylation-deficient mutant of prM reduces the secretion of E protein to 60% in comparison to the wild-type prM. Further evidence for the crucial role of prM glycan was described in the case of WNV. The prM glycosylation-deficient mutants decreased the formation and release of virus-like particles as well as genome copies. However, the infectivity of prM glycosylation-deficient mutants was not affected in mosquito, avian or mammalian cell lines [284].

In summary, *N*-linked glycosylation of TBEV prM and E proteins represents a multifaceted factor which is involved in many steps of the viral life-cycle, especially in virion assembly/secretion, and host/vector competence. Despite various studies, there are many aspects which need to be elucidated, especially the role of viral protein *N*-linked glycans within tick vectors. Moreover, the presence of other *N*-linked glycans in predicted sites of NS1, NS3, NS4B, and NS5 remains to be determined as well as their potential function.

## **Conclusions**

The recent decades have provided an outstanding amount of new data about glycoconjugates and a growing line of evidence highlights the importance of carbohydrate-based interactions in the complex pathogen-host environment [288]. Glycoconjugates have an enormous structural diversity in the glycan moieties and therefore fulfil a variety of biological roles [289]. Given the fact that glycoconjugates are the major components of the outer surface of animal cells [290], it is likely that all interactions of microbial pathogens with their hosts/vectors are affected to a certain degree by the pattern of glycans and glycan-binding molecules that each produces. Despite the fact that protein glycosylation in the field of tick-borne pathogens has become a subject of increased attention in the last decade [37, 218, 291], there is still a deep knowledge gap regarding the nature and the specific roles of the glycoconjugates in the infectious cycle of these pathogens. All the findings mentioned in this review have tackled the important, yet still inadequately explored, the field of the carbohydrate-based interactions at the pathogen-tick-host interface. The basis of these interactions needs to be further addressed to gain clearer insights into the intricate strategies that the parasites employ to successfully finish their life-cycles. Ultimately, the common goal of scientists working in any field dealing with infectious diseases is to find an effective countermeasure against the particular threat. Ticks transmit a great variety of bacterial, viral and protozoan pathogens and therefore the search for a potent vaccine against each of these parasites costs an enormous amount of effort and money. One of the most promising strategies to cope with all pathogens transmitted by ticks is the development of a general anti-tick vaccine [292]. The potentially important role of sugar moieties in such a tick vaccine has already been suggested, showing, for instance, the tick midgut protein Bm86 to be more immunogenic in glycosylated form than non-glycosylated [291]. However, the progress in this matter is still insufficient and intense analysis of glycosylation needs to be addressed in future studies in order to be applied to the development of new therapeutics. Modern glycan sequencing technologies and strategies that allow site-specific mass-spectrometric identification of proteins with glycan modifications in a complex biological sample have shown that glycosylation could be much more extensive than previously thought [293].

## **Abbreviations**

ACG: Cholesteryl 6-O-acyl-β-D-galactopyranoside
ACGal/Bb-GL-1: Cholesteryl 6-O-palmitoyl-β-D-galactopyranoside
AmOmp: *Anaplasma marginale* outer membrane protein
AP: Alternative complement pathway
ApOmp: *Anaplasma phagocytophilum* outer membrane protein
Bgp: *Borrelia* GAG-binding protein
CLR: C-type lectin receptor
CP: Classical complement pathway
CRASP: Complement regulator-acquiring surface protein
CRT: Calreticulin
DAF: Decay accelerating factor
DbpA: Decorin-binding protein
DENV: Dengue virus
ECM: Extracellular matrix
Erp: OspE/F-related proteins
FH: Complement factor H
FHL: Factor H-like
Fn: Fibrinogen
FREP: Fibrinogen-related protein
Fuc: Fucose
GAG: Glycosaminoglycan
GalCer: Galactosylceramide
IAFGP: *Ixodes scapularis* antifreeze glycoprotein
ISDLP: *Ixodes scapularis* dystroglycan-like protein
LacCer: Lactosylceramide
LP: Lectin complement pathway
LPS: Lipopolisaccharides
MAC: Membrane attack complex
MBL: Mannose-binding lectin
MGalD: Mono-α-galactosyl-diacylglycerol
MSP: Major surface proteins
MW: Molecular weight
na: Not available
NKT: Natural killer T cells
NLR/NOD-like receptors: Nucleotide-oligomerization domain-like receptors
Omp: Outer membrane protein
Osp: Outer surface protein
PAMP: Pathogen-associated molecular pattern
PM: Peritrophic matrix
PRR: Pattern recognition receptor
PSGL–1: P-selectin glycoprotein ligand-1
RGD: Arg-Gly-Asp tripeptide, a binding motif of fibronectin
Salp: Salivary protein
SG: Salivary glands
Sia: Sialic acid
TBEV: Tick-borne encephalitis virus
TLR: Toll-like receptors
TROSPA: Tick receptor for OspA
TSLPI: Tick salivary lectin pathway inhibitors
Vmp: Variable major protein
Vsp: Variable small protein
Vtp: Variable tick protein
WNV: West Nile virus
